# Supplementary material for: The causal relationship between the human gut microbiota and pyogenic arthritis: a Mendelian randomization study
Source: Front Cell Infect Microbiol. 2024 Nov 26;14:1452480. doi: 10.3389/fcimb.2024.1452480 (PMC11629706; doi:10.3389/fcimb.2024.1452480)
Supplement: Supplementary file 1 [file DataSheet1.zip › mendelian test/ebi-a-GCST90027742.csv_leave_one_out.pdf]

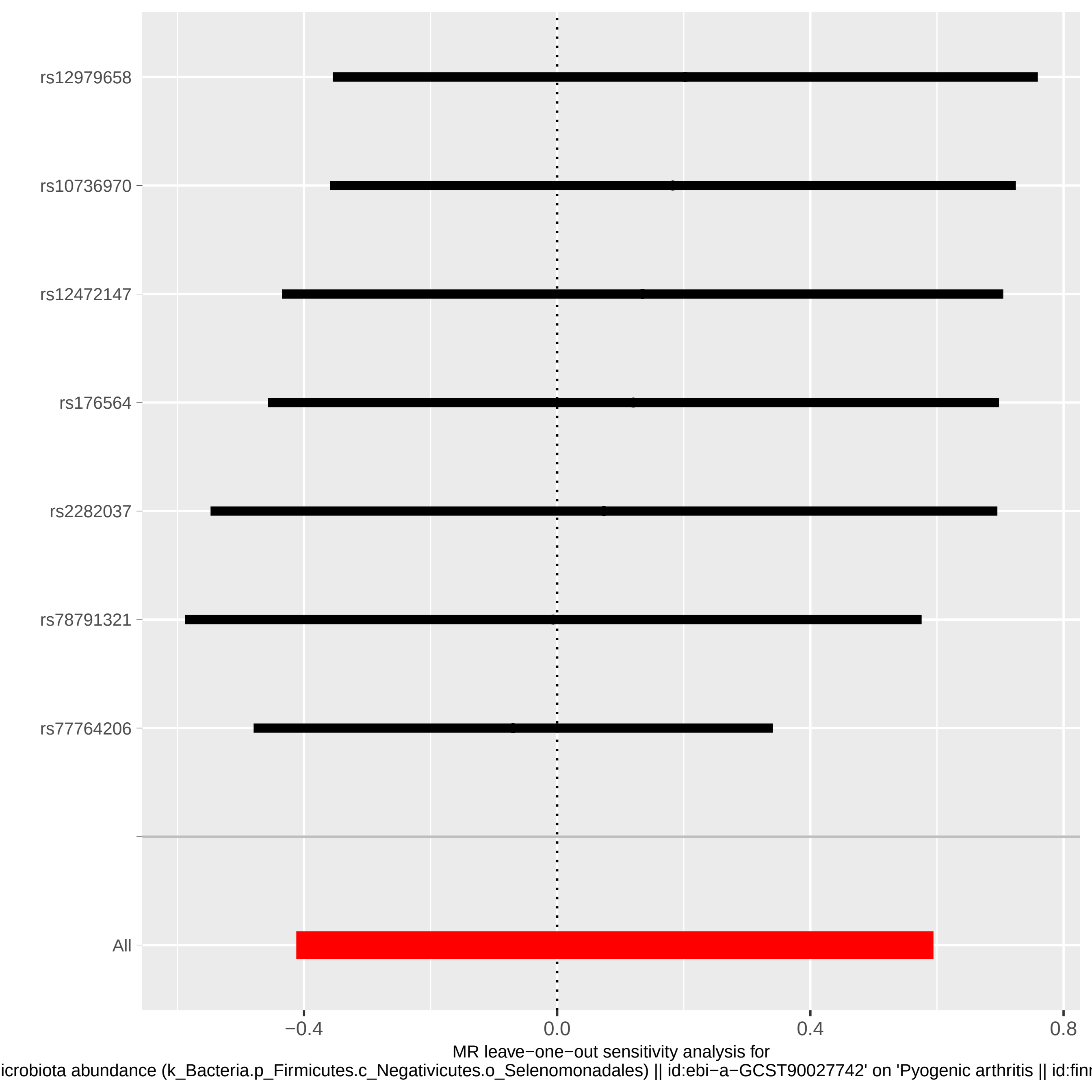
rs12979658
rs10736970
rs12472147
rs176564
rs2282037
rs78791321
rs77764206
All
−0.4
0.0
0.4
0.8
MR leave−one−out sensitivity analysis for
icrobiota abundance (k_Bacteria.p_Firmicutes.c_Negativicutes.o_Selenomonadales) || id:ebi−a−GCST90027742' on 'Pyogenic arthritis || id:finn
